# Hypoxic Preconditioning Enhances the Potential of Mesenchymal Stem Cells to Treat Neonatal Hypoxic-Ischemic Brain Injury

**DOI:** 10.1161/STROKEAHA.124.048964

**Published:** 2025-04-18

**Authors:** Sara T. De Palma, Eva C. Hermans, Tatiana M. Shamorkina, Chloe Trayford, Sabine van Rijt, Albert J.R. Heck, Cora H.A. Nijboer, Caroline G.M. de Theije

**Affiliations:** Department for Developmental Origins of Disease, University Medical Center Utrecht Brain Center and Wilhelmina Children’s Hospital (S.T.D.P., E.C.H., C.H.A.N., C.G.M.d.T.), Utrecht University, the Netherlands.; Biomolecular Mass Spectrometry and Proteomics, Bijvoet Center for Biomolecular Research and Utrecht Institute for Pharmaceutical Sciences, (T.M.S., A.J.R.H.), Utrecht University, the Netherlands.; Netherlands Proteomics Center, Utrecht, the Netherlands (T.M.S., A.J.R.H.).; Department of Instructive Biomaterials Engineering, MERLN Institute for Technology-Inspired Regenerative Medicine, Maastricht University, the Netherlands (C.T., S.v.R.).

**Keywords:** brain injuries, hypoxia-ischemia, brain, mesenchymal stem cells, neonate, neurogenesis, proteomics

## Abstract

**BACKGROUND:**

Neonatal hypoxic-ischemic (HI) brain injury is one of the leading causes of long-term neurological morbidity in newborns. Current treatment options for HI brain injury are limited, but mesenchymal stem cell (MSC) therapy is a promising strategy to boost neuroregeneration after injury. Optimization strategies to further enhance the potential of MSCs are under development. The current study aimed to test the potency of hypoxic preconditioning of MSCs to enhance the therapeutic efficacy in a mouse model of neonatal HI injury.

**METHODS:**

HI was induced on postnatal day 9 in C57Bl/6 mouse pups. MSCs were cultured under hypoxic (hypoxic-preconditioned MSCs [HP-MSCs], 1% O_2_) or normoxic-control (normoxic-preconditioned MSCs [NP-MSCs], 21% O_2_) conditions for 24 hours before use. At 10 days after HI, HP-MSCs, NP-MSCs, or vehicle were intranasally administered. Gold nanoparticle–labeled MSCs were used to assess MSC migration 24 hours after intranasal administration. At 28 days post-HI, lesion size, sensorimotor outcome, and neuroinflammation were assessed by hematoxylin and eosin staining, cylinder rearing task, and ionized calcium-binding adapter molecule 1 (IBA1) staining, respectively. In vitro, the effect of HP-MSCs was studied on transwell migration, neural stem cell differentiation and microglia activation, and the MSC intracellular proteomic content was profiled using quantitative Liquid Chromatography-Tandem Mass Spectrometry (LC-MS/MS).

**RESULTS:**

Intranasally administered HP-MSCs were superior to NP-MSCs in reducing lesion size and sensorimotor impairments post-HI. Moreover, hypoxic preconditioning enhanced MSC migration in an in vitro set-up, and in vivo to the lesioned hemisphere after intranasal application. In addition, HP-MSCs enhanced neural stem cell differentiation into more complex neurons in vitro but had similar anti-inflammatory effects compared with NP-MSCs. Lastly, hypoxic preconditioning led to elevated abundances of proteins in MSCs related to extracellular matrix remodeling.

**CONCLUSIONS:**

This study shows for the first time that hypoxic preconditioning enhanced the therapeutic efficacy of MSC therapy in a mouse model of neonatal HI brain injury by increasing the migratory and neuroregenerative capacity of MSCs.

Neonatal hypoxic-ischemic (HI) brain injury, caused by perinatal asphyxia, is a major contributor to mortality and neurological morbidity in neonates born at term.^1^ The incidence of HI brain injury ranges from 1.6 up to 12.1 per 1000 live-births in high-income and developing countries, respectively.^1^ Out of all neonates with moderate to severe HI brain injury, respectively 30% and 75% develop long-term disabilities including motor, cognitive, and behavioral problems.^2^ Therapeutic hypothermia is the only effective clinical treatment currently available for neonates with perinatal asphyxia.^3^ However, therapeutic hypothermia only provides partial protection in moderate and severe cases of perinatal asphyxia and has a limited treatment window (within 6 hours after the insult),^3^ highlighting the need for novel therapies, especially those with a longer therapeutic window.

Regenerative cell–based strategies to repair the injured neonatal brain provide a promising alternative approach. Recently, our group demonstrated the feasibility and safety of intranasal mesenchymal stem cell (MSC) therapy in infants with HI brain injury caused by perinatal stroke.^4^ In addition, intranasal MSC therapy effectively reduces lesion size and improves long-term sensorimotor and cognitive outcome in experimental models for neonatal brain injury.^5–8^ Upon intranasal administration, MSCs migrate specifically to the lesion site and start to disappear within 24 hours,^6^ indicating that MSCs in the brain are short-lived and do not integrate into the brain parenchyma. At the lesion site, MSCs respond to the HI microenvironment by secreting various trophic and immunomodulatory factors that can tweak the environment into a pro-repair state thereby boosting neurogenesis and dampening neuroinflammation.^9^

Intranasal MSC therapy in rodent models reduces lesion size by ±30% to 50%,^5,8^ leaving a window to improve the therapeutic efficacy. A promising optimization strategy is the hypoxic preconditioning of MSCs prior to administration. Hypoxic preconditioning has been shown to enhance the therapeutic potential of MSCs in various models of ischemic pathological conditions, including cerebral ischemia, stroke, hindlimb ischemia, and acute kidney injury.^10–13^ Previous studies showed that culturing MSCs at 1% to 5% O_2_ levels results in upregulation of transcription factor Hif1α (hypoxia-inducible factor 1 alpha),^10^ which in turn increases Cxcr4 (C-X-C chemokine receptor type 4) and Mmp9 (matrix metalloproteinase-9) expression, enhancing the migratory capacity and survival of MSCs.^10,13^ Furthermore, hypoxic preconditioning improves the effects of MSCs on neuronal survival, likely by enhanced secretion of neurotrophic factors, such as Bdnf (brain-derived neurotrophic factor).^12^ Importantly, extracellular vesicles and conditioned medium from hypoxic-preconditioned MSCs (HP-MSCs) exert similar effects to intact HP-MSCs, indicating a large role for the secretome in the superior efficacy after hypoxic preconditioning.^14^ While increased therapeutic potency of HP-MSC therapy has been demonstrated in adult models of brain injury, the benefits for the injured neonatal brain remain unstudied. Therefore, the aim of this study was to assess the potential superior effects of HP-MSC therapy in a mouse model for neonatal HI brain injury. Additionally, we explored mechanisms of action of hypoxic preconditioning by examining the MSC migratory capacity in vivo and in vitro, the effects on neuroinflammation in vivo and in vitro, and the effects on neurogenesis in vitro. Moreover, we assessed differentially expressed proteins in HP-MSCs compared with normoxic-preconditioned MSCs (NP-MSCs) to shed more light on the underlying mechanisms of this optimization strategy.

## Methods

### Availability of Supporting Data

The mass spectrometry proteomics data have been deposited to the ProteomeXchange Consortium via the Proteomics Identifications Database (PRIDE)^15^ partner repository with the data set identifier PXD053838.

The rest of the data are available from the corresponding author upon reasonable request.

### Ethical Approval and Reporting Guidelines

All procedures were performed according to the Dutch and European international guidelines (Directive 86/609, ETS 123, Annex II), and were approved by the Central Authority for Scientific Procedures on Animals (The Hague, the Netherlands; approval number: AVD115002016751) and the local Experimental Animal Committee Utrecht (Utrecht University, Utrecht, the Netherlands). The experiments are reported in compliance with the ARRIVE guidelines (Animal Research: Reporting of In Vivo Experiments).^16^

### Animal Model

HI injury was induced in 9-day-old pups by permanent unilateral carotid artery ligation under isoflurane anesthesia (5–10 minutes; 5% induction, 3% to 4% maintenance with flow O_2_: air 1:1), followed by recovery with their mother for at least 75 minutes and subsequently systemic hypoxia at 10% O_2_ for 45 minutes in a temperature-controlled and humidified hypoxic incubator. Detailed materials and methods are available in the Supplemental Material and an overview of the number of animals is presented in Table S1.

### Statistical Analysis

In in vivo and in vitro analyses, the experimental units were individual mice and individual wells, respectively. Statistical analysis and visualization of data were performed by using GraphPad Prism 9.3 and Rstudio (Rstudio, PBC). Data were checked for normal gaussian distribution and significant outliers were detected by Robust Regression and Outlier Removal (ROUT) outlier method (*Q*=1%). Unpaired *t* test (2 groups) or 1-way ANOVA followed by Holm-Šidàk post hoc tests (>2 groups) were performed. Raw data are shown as mean±SD. Results were considered statistically significant if *P*<0.05. For proteomics analysis, see Supplemental Material.

## Results

### Hypoxic Preconditioning Enhances the Therapeutic Efficacy of Intranasal MSC Therapy by Reducing Lesion Size and Sensorimotor Impairments After Neonatal HI

**Figure 1.**
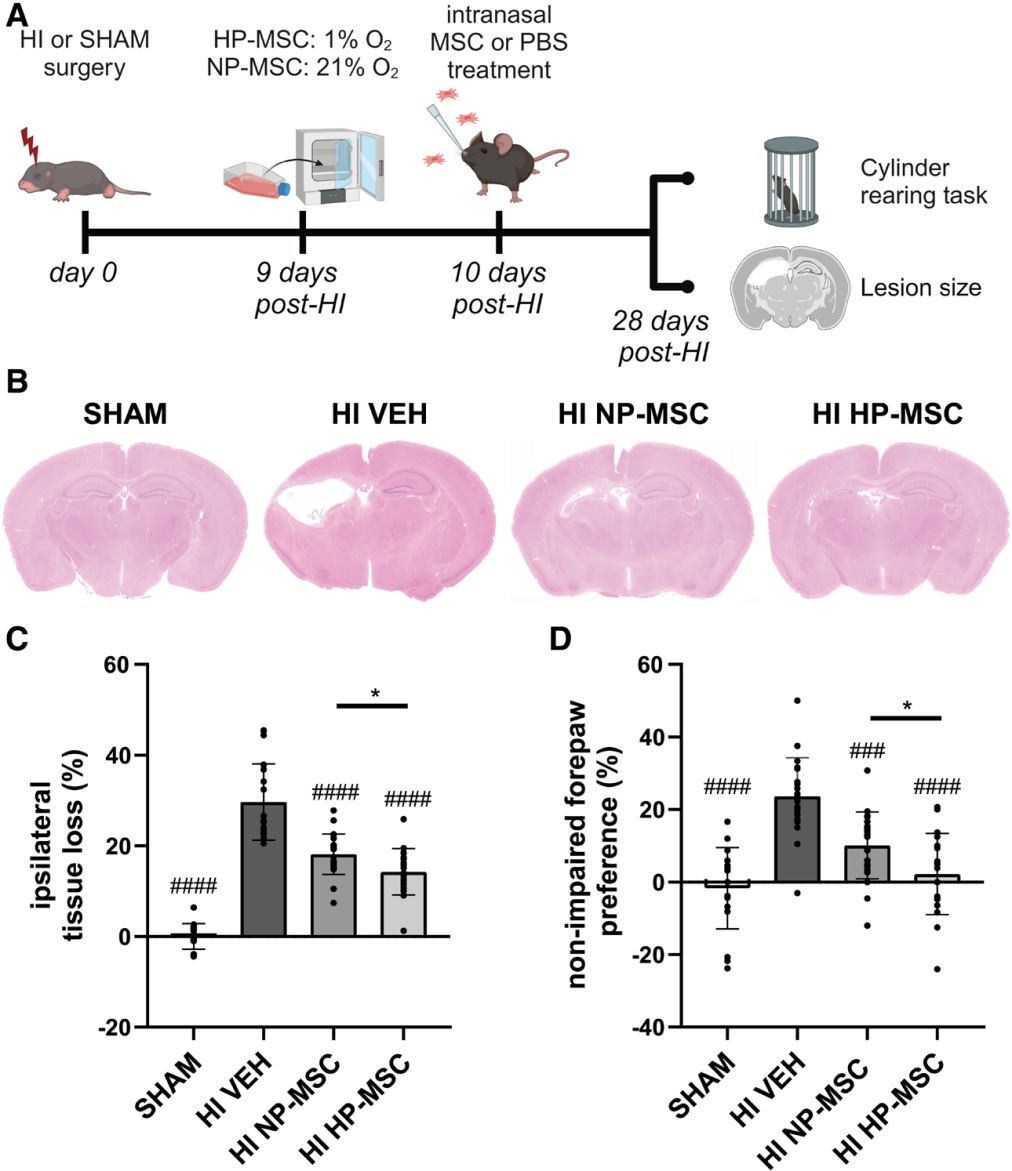
**Superior effect of hypoxic-preconditioned mesenchymal stem cells (HP-MSCs) on lesion size and functional outcome in a mouse model of neonatal hypoxic-ischemic (HI) brain injury. A**, Overview of study design. **B**, Representative images of ipsilateral tissue loss visualized by hematoxylin and eosin staining in SHAM-control mice or HI-injured mice intranasally treated with either vehicle (VEH), normoxic-preconditioned MSCs (NP-MSCs), or HP-MSCs at 10 days post-HI. **C**, Quantification of ipsilateral tissue loss (%) at 28 days post-HI. **D**, Non-impaired forepaw preference at 28 days post-HI; SHAM: n=18, HI-VEH: n=21, HI–NP-MSC (**C**): n=23; NP-MSC (**D**): n=22, HI–HP-MSC: n=19. Data represent mean±SD. ###*P*<0.001, ####*P*<0.0001 significance relative to HI-VEH; *P<0.05 HI–HP-MSC vs HI–NP-MSC.

To investigate whether hypoxic preconditioning enhances the efficacy of intranasal MSC therapy, HP-MSCs, NP-MSCs, or vehicle solution were given intranasally to C57Bl/6 mice at 10 days after induction of HI. Lesion size and sensorimotor impairment were assessed at 28 days post-HI (Figure 1A). Because a 2-way ANOVA revealed no significant effect of sex in any of the groups (SHAM female [F] versus male [M]: *P*=0.8642; HI-vehicle F versus M: *P*=0.4299; HI NP-MSC F versus M: *P*=0.8487; HI HP-MSC F versus M: *P*=0.8642; data not shown), males and females where analyzed as 1 group. Vehicle-treated HI mice showed ≈30% tissue loss of the ipsilateral hemisphere compared with SHAM-control mice (*P*<0.0001; Figure 1B and 1C; Figure S1). Intranasal NP-MSC treatment significantly reduced tissue loss in the ipsilateral hemisphere of HI-injured mice compared with vehicle treatment (*P*<0.0001). HP-MSCs were significantly more effective in reducing tissue loss compared with NP-MSCs (*P*=0.0307).

In addition, vehicle-treated HI mice showed a preference to use the non-impaired forepaw in the cylinder rearing task, indicative of reduced sensorimotor function, compared with SHAM-control mice (*P*<0.0001, Figure 1D). Treatment with NP-MSCs significantly improved sensorimotor function compared with vehicle-treated mice (*P*=0.0001) but hypoxic preconditioning significantly enhanced the therapeutic effect of MSCs on sensorimotor impairments compared with NP-MSCs (*P*=0.0193).

### Hypoxic Preconditioning Enhances Migration of MSCs Toward the Lesion Site

**Figure 2.**
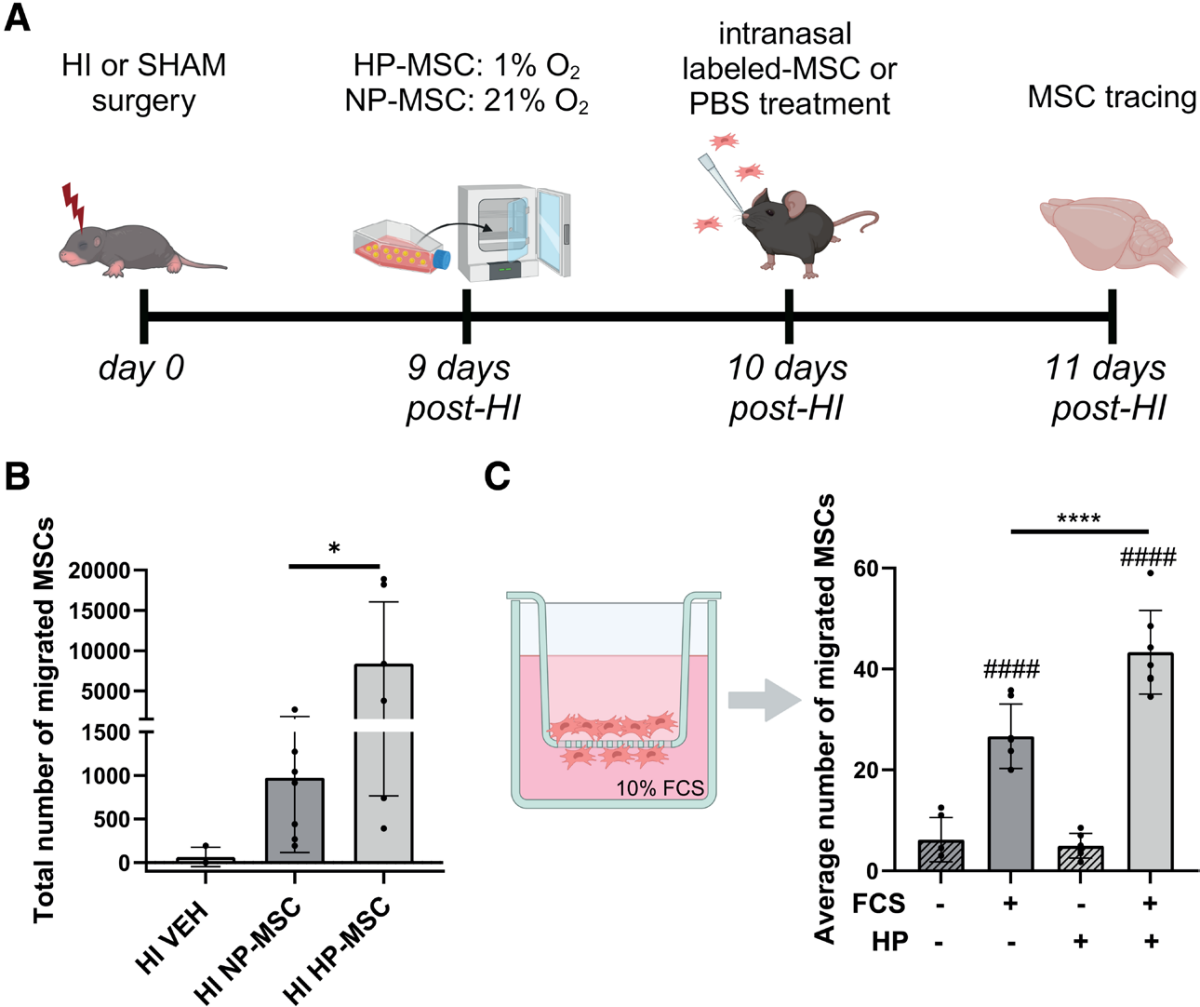
**Enhanced migratory capacity of hypoxic-preconditioned mesenchymal stem cells (HP-MSCs) compared with normoxic-preconditioned MSCs (NP-MSCs) in vivo and in vitro. A**, Overview of the study design. **B**, Gold-labeled MSC migration toward the ipsilateral lesioned hemisphere expressed in total number of cells; hypoxic-ischemic (HI)–vehicle (VEH): n=3, HI–NP-MSC: n=7, HI–HP-MSC: n=7. The HI-VEH group was used as negative control only and was not included in the statistical analysis. **C**, **left**, schematic overview of transwell migration assay; **right**, quantification of number of HP-MSCs or NP-MSCs migrated toward 10% fetal calf serum (FCS) in vitro; n=6–7 per condition. Data represent mean±SD. *P<0.05; ****P<0.0001 HP-MSCs vs NP-MSCs, ####*P*<0.0001 relative to no FCS condition.

The superior therapeutic effect of HP-MSCs may be attributed to an increased migratory capacity of HP-MSCs towards the HI-injured brain. Therefore, the migratory capacity was assessed by intranasal administration of gold nanoparticle–labeled MSCs^17^ (Figure 2A). Unpaired *t* test showed that significantly more labeled HP-MSCs migrated towards the injured hemisphere compared with NP-MSCs (*P*=0.0251; Figure 2B). HI-vehicle group was used as a control. The increased migratory capacity of MSCs after hypoxic preconditioning was confirmed in an in vitro transwell migration assay. Both NP-MSCs and HP-MSCs migrated significantly to 10% fetal calf serum (FCS) compared with no FCS (*P*<0.0001), however hypoxic preconditioning significantly increased the migratory capacity of MSCs in vitro (*P*<0.0001; Figure 2C).

### Hypoxic Preconditioning Does Not Enhance the Anti-Inflammatory Effects of the MSC Secretome

**Figure 3.**
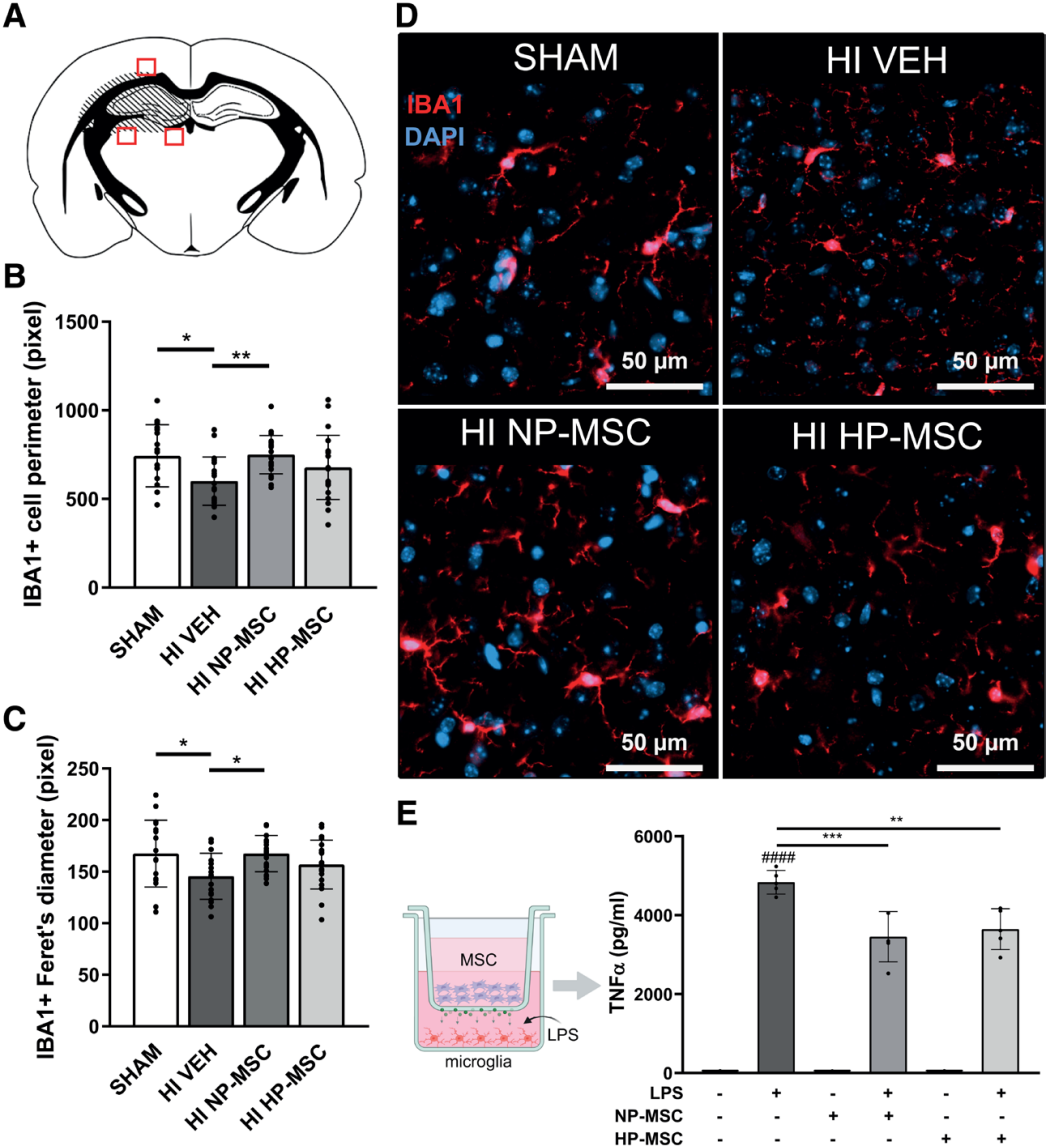
**Effect of hypoxic-preconditioned mesenchymal stem cells (HP-MSCs) on microglia activation after hypoxic-ischemic (HI) injury in vivo and lipopolysaccharide (LPS) exposure in vitro. A**, Perilesional locations of images taken in ionized calcium-binding adapter molecule 1 (IBA1)-stained brain sections at P28. **B**, Quantification of perimeter of IBA1^+^ cells. **C**, Quantification of Feret diameter of IBA1^+^ cells. **D**, Representative fluorescent images of perilesional IBA1^+^ cells (40×) in SHAM controls, HI-vehicle (VEH), HI–normoxic-preconditioned MSC (NP-MSC), and HI–HP-MSC mice. **E**, **left**, schematic overview of non-contact co-culture of primary-isolated mouse microglia exposed to 50 ng/mL LPS with NP-MSCs or HP-MSC (purple cells) in the hanging insert, green dots represent MSC secretome that can reach the medium of the microglia; **right**, TNF-α (tumor necrosis factor-α) secretion by microglia after 24-hour exposure to LPS. Data represent mean±SD. **B** and **C**, SHAM: n=17, HI-VEH: n=19, HI–NP-MSC: n=22, and HI–HP-MSC: n=21. **E**, n=5 per condition. DAPI indicates 4',6-diamidino-2-phenylindole. *P<0.05, **P<0.01, ***P<0.001 between indicated groups, ####*P*<0.0001 compared with non-LPS condition.

To further dive into the enhanced potency of HP-MSCs, we examined whether hypoxic preconditioning enhanced the anti-inflammatory capacity of MSCs. In vivo, the effects of intranasal HP-MSCs and NP-MSCs administration on microglia activation, measured by the morphology of perilesional ionized calcium-binding adapter molecule 1 (IBA1)^+^ cells, was assessed at 28 days post-HI (Figure 3A). IBA1^+^ cells in HI-vehicle mice showed a smaller perimeter (*P*=0.0179) and a smaller Feret's diameter (*P*=0.0227) compared with SHAM littermates, indicating a more ameboid (activated) shape (Figure 3B and 3D). NP-MSC treatment successfully mitigated the activation state of IBA1^+^ cells compared with vehicle treatment (Figure 3B: *P*=0.0094; Figure 3C: *P*=0.0188). HP-MSCs did not significantly attenuate microglia activation compared with vehicle (Figure 3B: *P*=0.2112; Figure 3C: *P*=0.2624), although no significant difference between HP-MSCs and NP-MSCs was observed (Figure 3B: *P*=0.2112; Figure 3C: *P*=0.2624). No changes in microglia morphology were detected in the contralateral hemispheres of HI animals in all experimental groups (Figure S2). In vitro, non-contact co-culture with both NP-MSCs and HP-MSCs significantly reduced the secretion of proinflammatory TNF-α (tumor necrosis factor-α) by lipopolysaccharide (LPS)-exposed microglia compared with untreated LPS-exposed microglia to a similar extent (*P*=0.0007 and *P*=0.0017, respectively, Figure 3E), indicating that hypoxic preconditioning does not enhance the anti-inflammatory capacity of the MSC secretome as such.

### Hypoxic Preconditioning of MSCs Enhances Their Supportive Effects on Neural Stem Cell Differentiation

**Figure 4.**
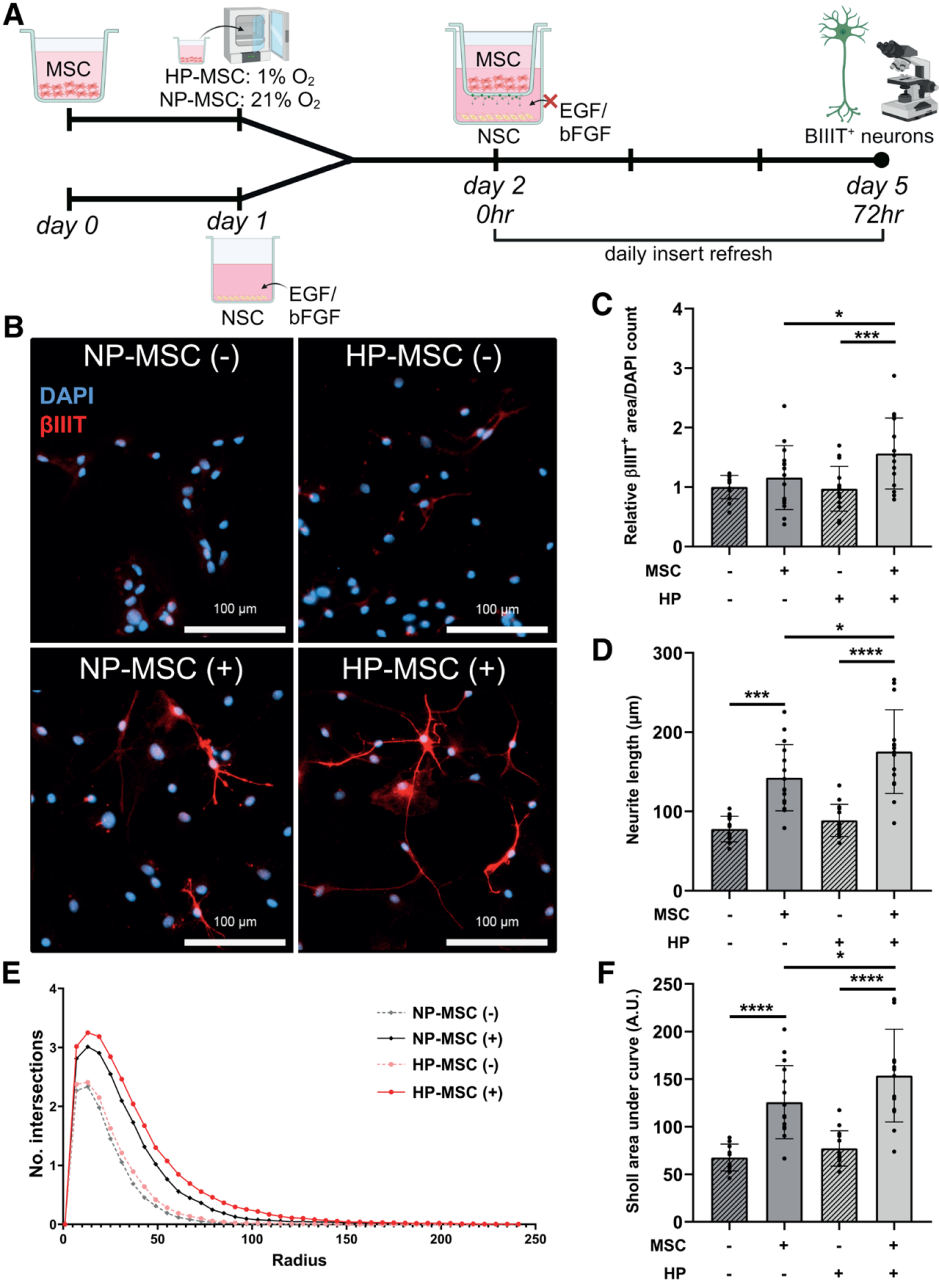
**Hypoxic-preconditioned mesenchymal stem cells (HP-MSCs) enhance differentiation of neural stem cells (NSCs) into more complex neurons than normoxic-preconditioned MSCs (NP-MSCs). A**, Overview of experimental design. **B**, Representative fluorescent images (10×) of βIIIT (βIII-tubulin)^+^ cells (differentiated NSCs) in co-culture with NP-MSCs or HP-MSCs (+) or empty inserts (−). **C**, Quantification of βIIIT^+^ area normalized to the cell numbers (4',6-diamidino-2-phenylindole [DAPI] count) relative to the NP-MSCs (−) condition. **D**, Quantification of neurite length of βIIIT^+^ neurons. **E**, Number of intersections of βIIIT^+^ neurons with circles of increasing radius in Sholl analysis. **F**, Quantification of Sholl analysis by area under the curve. Data represent mean±SD. All: n=13–15 per condition out of 3 independent experiments. A.U. indicates arbitrary unit; bFGF, basic fibroblast growth factor; and EGF, epidermal growth factor. *P<0.05, ***P<0.001, and ****P<0.0001.

Next, the effect of hypoxic preconditioning on enhancing the neuro-supportive capacity of MSCs was assessed in vitro in a non-contact co-culture of MSCs with differentiating neural stem cells (NSCs). At 72 hours after the onset of NSC differentiation (Figure 4A), Nestin^+^ area, as a measure of undifferentiated NSCs, was significantly decreased confirming successful differentiation (*P*<0.0001; Figure S3). Differentiated NSCs co-cultured with HP-MSCs showed a higher βIII-tubulin^+^ area compared with NSCs co-cultured with NP-MSCs (*P*=0.0197, Figure 4B and 4C), suggesting HP-MSCs have a superior boosting effect on the formation of neurons. Furthermore, morphometric analysis of the βIII-tubulin^+^ neurons showed that co-culture with HP-MSCs induced the formation of neurons with longer neurites (*P*=0.0113; Figure 4D) and higher branch complexity (*P*=0.0256; Figure 4E and 4F) compared with those differentiated in co-culture with NP-MSCs. Altogether, hypoxic preconditioning enhanced the potency of the MSC secretome to promote NSC differentiation into more complex neurons. To confirm the secretome of HP-MSCs was responsible for the formation of more complex neurons, NSCs were differentiated in the presence of MSC–conditioned medium only (no MSCs) for 72 hours. HP-MSC–conditioned medium induced NSCs differentiation into βIIIT^+^ neurons with longer neurites (*P*=0.0002) and higher branch complexity (*P*=0.0002) compared with NP-MSC–conditioned medium (Figure S4), confirming that hypoxic preconditioning optimized the MSC secretome to drive neurogenesis.

### Hypoxic Preconditioning Alters the Intracellular Proteome Profile of MSCs Toward a Promigratory State

**Figure 5.**
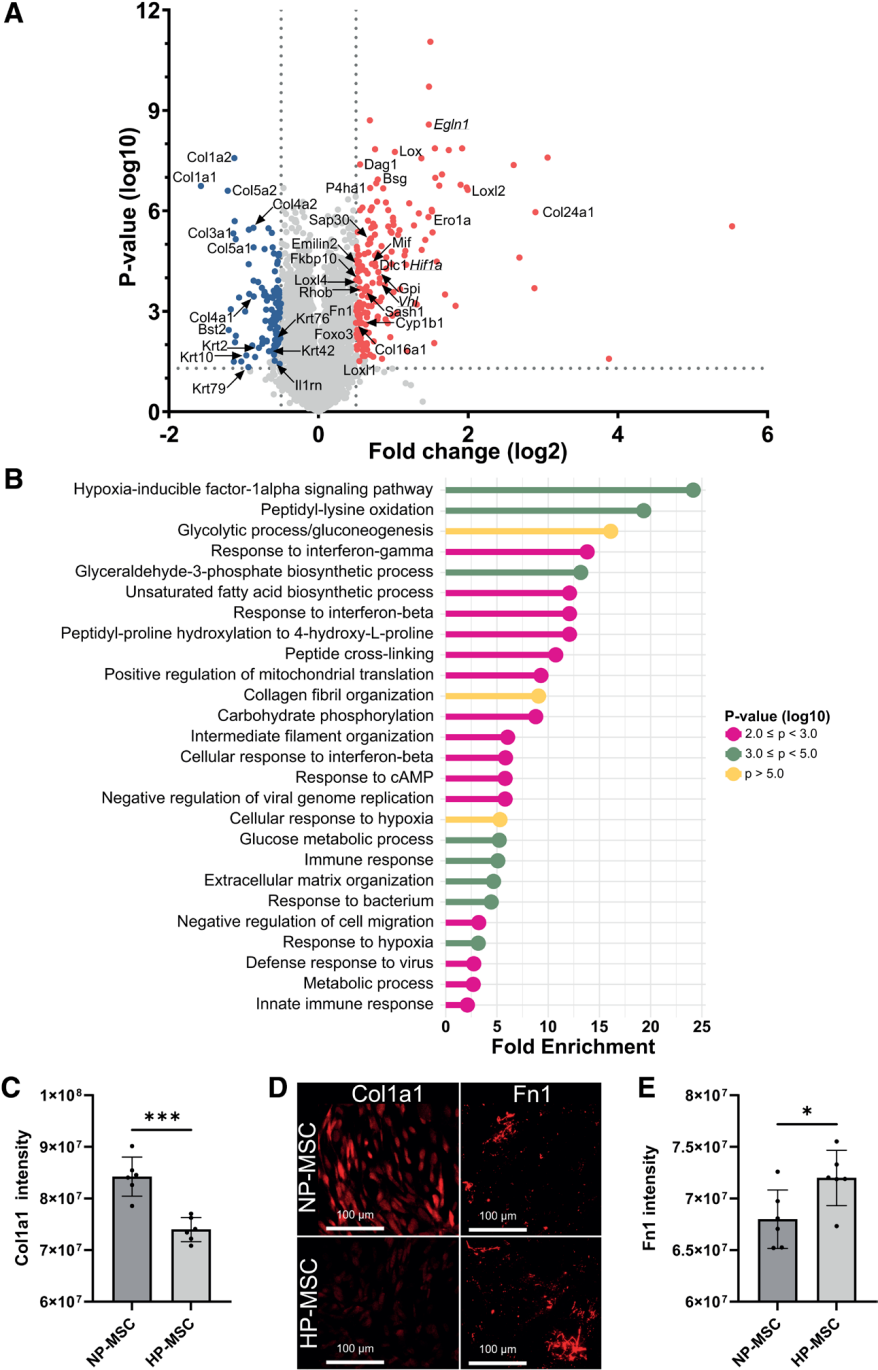
**Hypoxic preconditioning of mesenchymal stem cells (MSCs) leads to increased intracellular expression of Hif1α (hypoxia-inducible factor 1 alpha) and proteins primarily involved in glucose metabolism and ECM (extracellular matrix) remodeling. A**, Volcano plot with significantly upregulated (red) and downregulated (blue) proteins directly after hypoxic preconditioning. Annotated proteins are found to be involved in migratory processes by enrichment analysis. Hif1α and its interactors are underlined; n=2 per condition. **B**, Pathway enrichment analysis of upregulated and downregulated proteins in hypoxic-preconditioned MSCs (HP-MSCs) vs normoxic-preconditioned MSCs (NP-MSCs). **C**, Quantification of Col1a1 (collagen alpha-1(I) chain) expression by NP-MSCs and HP-MSCs. **D**, Representative fluorescent images (10×) of Fn1 (fibronectin 1)^+^ ECM and Col1a1^+^ MSCs in vitro. **E**, Quantification of Fn1 expression in the ECM of NP-MSCs and HP-MSCs. Data represent mean±SD. **C** and **E**, n=6 per condition. Bsg indicates basigin; Bst2, Bone marrow stromal antigen 2; Col16a1, collagen alpha-1(XVI) chain; Col1a1, collagen alpha-1(I) chain; Col1a2, collagen alpha-2(I) chain; Col24a1, collagen alpha-1(XXIV) chain; Col3a1, collagen alpha-1(III) chain; Col4a1, collagen alpha-1(IV) chain; Col4a2, collagen alpha-2(IV) chain; Col5a1, collagen alpha-1(V) chain; Col5a2, collagen alpha-2(V) chain; Cyp1b1, cytochrome P450 1B1; Dag1, dystroglycan 1; Dlc1, Rho GTPase-activating protein 7; Egln1, Egl [egg-laying defective] nine homolog 1; Ero1a, ERO1-like protein alpha; Fkbp10, peptidyl-prolyl cis-trans isomerase FKBP10; Foxo3, Forkhead box protein O3; Gpi, glucose-6-phosphate isomerase; Il1rn, interleukin-1 receptor antagonist protein; Krt10, keratin, type I cytoskeletal 10; Krt2, keratin, type II cytoskeletal 2 epidermal; Krt42, keratin, type I cytoskeletal 42; Krt5, keratin, type II cytoskeletal 5; Krt76, keratin, type II cytoskeletal 2 oral; Krt79, keratin, type II cytoskeletal 79; Lox, protein-lysine 6-oxidase; Loxl1, lysyl oxidase homolog 1; Loxl2, Lysyl oxidase homolog 2; Loxl4, Lysyl oxidase homolog 4; Mif, Macrophage migration inhibitory factor; P4ha1, prolyl 4-hydroxylase subunit alpha-1; Rhob, Rho-related GTP-binding protein RhoB; Sap30, histone deacetylase complex subunit SAP30; Sash1, SAM and SH3 domain-containing protein 1; and Vhl, Von Hippel–Lindau tumor suppressor. *P<0.05, ***P<0.001.

To shed more light on the superior actions of HP-MSCs, we examined intracellular changes induced by hypoxic preconditioning in MSCs by proteome analysis immediately after hypoxic preconditioning. A total of 309 proteins were found to be significantly altered by hypoxic preconditioning in comparison to NP-MSCs (Figure 5A; volcano plot showing significantly upregulated [red] and downregulated [blue] proteins directly after hypoxic preconditioning). As expected, Hif1α and its responsive proteins Egln1 (Egl [egg-laying defective] nine homolog 1) and Vhl (Von Hippel–Lindau tumor suppressor) were upregulated in HP-MSCs compared with NP-MSCs (Hif1a: Log_10_[*P*]=4.39; Egln1: Log_10_[*P*]*=*8.58; Vhl: Log_10_[*P*]=3.85), validating the hypoxic procedure. Accordingly, pathway enrichment analysis revealed that hypoxic preconditioning enhanced the modulation of hypoxia-responsive genes, as well as glucose metabolism and ECM (extracellular matrix) remodeling (Figure 5B; Table S2). Importantly, hypoxic preconditioning altered MSC intracellular expression of collagens (eg, Col24a1 [collagen alpha-1(XXIV) chain]: Log_10_[*P*]=5.96; Col1a1 [collagen alpha-1(I) chain]: Log_10_[*P*]=6.74), keratins (eg, Krt5 [keratin 5]: Log_10_[*P*]=1.43; Krt76 [keratin 76]: Log_10_[*P*]=2.14), lysyl oxidase proteins (eg, Loxl2 [lysyl oxidase like 2]: Log_10_[*P*]=6.63; Loxl4 [lysyl oxidase like 4]: Log_10_[*P*]=3.89), and other proteins (eg, Bsg [basigin]: Log_10_[*P*]=6.94; Fn1 [fibronectin 1]: Log_10_[*P*]=3.02; Il1rn [interleukin 1 receptor antagonist]: Log_10_[*P*]=1.52) associated with ECM-remodeling pathways that have been related to enhanced cell migratory capacity (Table 1). To confirm the effect of hypoxic preconditioning on ECM remodeling, the expression of Col1a1 and Fn1 was further investigated by immunocytochemistry. Our results showed that HP-MSCs express less intracellular Col1a1 (*P*=0.002; Figure 5C and 5D) and produce ECM that contains more Fn1 (*P*=0.0311; Figure 5D and 5E) in a similar pattern as indicated by the proteome analysis.

**Table 1.**
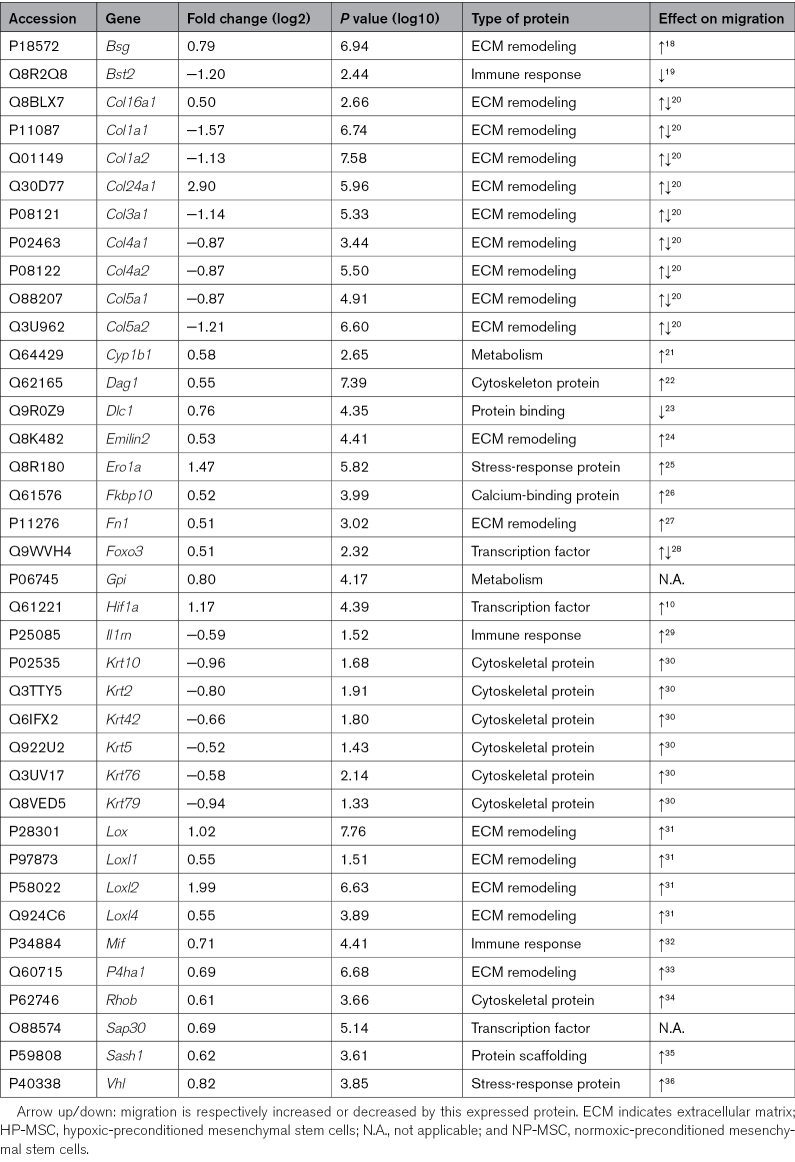
Upregulated and Downregulated Proteins in HP-MSCs Versus NP-MSCs Associated With Cell Migration–Related Pathways

### Hypoxic Preconditioning of MSCs Leads to Overexpression of Neurogenic Proteins

To investigate the enhanced neurogenic supportive potential of HP-MSCs over NP-MSCs, an extensive literature search was conducted, which revealed HP-MSCs upregulated 5 intracellular proteins with a secretome signature (i.e., that can be secreted), that are involved in neurogenic processes (Table 2). Particularly, Gpi (glucose-6-phosphate isomerase; Log_10_[*P*]=4.17), Sema3c (semaphorin-3c; Log_10_[*P*]=4.27) and Vldlr (very low-density lipoprotein receptor; Log_10_[*P*]=4.83) have been shown to promote axonal growth and guidance,^37–39^ whereas Hebp1 (heme-binding protein 1; Log_10_[*P*]=3.20) and Nampt (nicotinamide phosphoribosyltransferase; Log_10_[*P*]=6.27) are associated with neurovascular regenerative processes.^40,41^

**Table 2.**
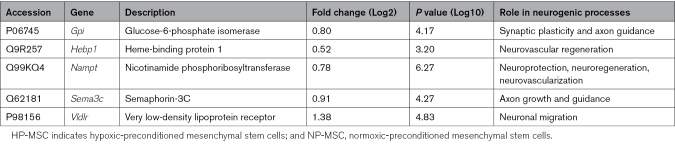
Intracellular Proteins With a Secretory Profile Reported to Be Involved in Neurogenic Processes and Upregulated in HP-MSCs Versus NP-MSCs

## Discussion

This study evaluated the potential of hypoxic preconditioning to enhance the treatment efficacy of intranasal MSC therapy. We demonstrated that hypoxic preconditioning of MSCs (1% O_2_ levels for 24 hours) before intranasal administration augments their therapeutic potential in a mouse model for neonatal HI brain injury. Although the beneficial effects of hypoxic preconditioning on bone marrow–derived MSCs have been shown for adult neurological diseases like stroke and intracerebral hemorrhage,^10–13^ the effect of hypoxic preconditioning of MSCs has not been shown on the neonatal injured brain, which is even more plastic and neurogenic than the adult brain. For the first time, we reported a global quantification of the total in vivo HP-MSC migration, whereas other studies only showed qualitative data or regional migration.^10,12^ Other studies have shown that hypoxic preconditioning can aid in the directional migration of MSCs by the upregulation of HIF1*α*^10^ via FAK signaling.^10,12^ Similarly, our proteomics data show that Hif1α protein and Hif1α-interactors Egln1 and Vhl are upregulated in MSCs after hypoxic preconditioning which is indicative of a clear activation of hypoxia pathways that can steer migration.^36^

Under hypoxic conditions, after Hif1α activation, cells are subjected to metabolic reprogramming towards glycolysis and consequently the deposition of lactate outside of the cell.^42^ Indeed, our proteomics analysis of HP-MSCs directly after preconditioning showed enrichment of pathways involved in glucose metabolism and ECM remodeling which aligns with studies of the proteome measured in hypoxic-preconditioned adipose-derived and bone marrow–derived MSCs.^43,44^ Particularly, we showed that hypoxic preconditioning altered the expression of multiple intracellular proteins in MSCs involved in ECM reorganization (eg, Krt42 and Dlc1 [deleted in liver cancer 1]) of which many are intracellular collagen-modifying proteins (eg, P4ha1 [prolyl 4-hydroxylase subunit alpha 1], Sash1 [SAM and SH3 domain containing 1], and Vhl), whereas Emilin2, Fn1, and Loxl2 are ECM-remodeling proteins that can be secreted outside the cell.^24,27,31^ In line with the proteomics data, our immunocytochemistry investigation of 2 important ECM proteins, Fn1 and Col1a, further supports the hypothesis that hypoxic preconditioning leads to a reorganization of the ECM. Overall, our data indicate that the increased migratory capacity of HP-MSCs may be driven by the production of proteins that aid in the remodeling of the ECM.

Once migrated to the lesion site, MSCs can have anti-inflammatory properties. In line with previous studies, we showed that NP-MSC treatment reduced microglia activation at 28 days post-HI.^6^ Hypoxic preconditioning of MSCs has been reported to enhance anti-inflammatory properties of MSCs in animal models of cerebral ischemia and intracerebral hemorrhage shortly after induction of injury.^11,45^ In our study, we observed that hypoxic preconditioning did not cause additional anti-inflammatory benefits to MSC therapy in vitro and in vivo. These observations were confirmed by the proteome analysis, which showed few pathways related to immune response processes were enriched in MSCs after hypoxic preconditioning, and the proteome contained both anti-inflammatory and pro-inflammatory proteins.

In the present study, we showed that hypoxic preconditioning boosted the neuroregenerative capacity of the MSC secretome by enhancing the neuronal complexity of differentiated NSCs in vitro. Here, we performed an intracellular proteome analysis of HP-MSCs and did not perform a full proteome analysis on the secretome, which could be performed in future studies to identify the secreted neurogenic proteins responsible for the enhanced neuroregenerative capacity of HP-MSCs. Nonetheless, we identified a set of intracellular proteins with a secretory profile that are upregulated after hypoxic preconditioning and which are involved in neurogenic processes (eg, Gpi, Sema3c, Vldlr, Hebp1, and Nampt).^37–41^ Moreover, our HP-MSCs modified the expression of collagens and fibronectins, ECM elements that can adapt ECM stiffness, thus possibly aiding in supporting neurite formation, managing trophic factor concentrations, and enhancing the endogenous migration of neural progenitors, which might explain the superior effect of HP-MSCs on neurorepair in vivo.^46^

To obtain maximum treatment efficacy in future clinical applications, future research should include studies to determine the optimal duration of hypoxic preconditioning of MSCs. In addition, given the anatomical differences between rodent and human olfactory pathways, it remains essential to confirm that (HP-)MSCs can migrate to the brain in experimental models closely resembling human babies. Importantly, clinical implementation of hypoxic preconditioning would require the adoption of hypoxic incubators in cell therapy facilities, posing potential logistic and regulatory challenges. Confirmatory studies on the magnitude of the therapeutic benefit, including various behavioral domains such as cognitive and social impairments, and in vitro potency assays for comparison strategies are needed to assess whether the clinical benefits outweigh the burden of integrating hypoxic preconditioning into the clinical setting.

In conclusion, this study shows that hypoxic preconditioning of MSCs is a promising and practical optimization strategy to enhance the therapeutic efficacy of intranasally applied MSCs for neonatal brain injury. Our results suggest that hypoxic preconditioning skews the phenotype of MSCs toward a more migratory-prone and neurorepair-supportive state. All in all, our study shows that hypoxic preconditioning could be a relatively easy, clinically applicable improvement strategy for MSC-based repair strategies to improve the prospects of vulnerable newborns with HI brain injury.

## Article Information

### Acknowledgments

The authors thank Erik van Tilborg, Caren van Kammen, Mirjam Maas, Rebecca Kleisen, Roeland E. Lokhorst, Youri Adolfs, Jeroen Pasterkamp, Ewout Groen, and the Laboratory of Translational Oncology for their excellent help during data collection. Illustrations were created using BioRender.com. Drs Nijboer and de Theije contributed to conceptualization, methodology, supervision, and funding acquisition. E.C. Hermans and S.T. De Palma contributed to the methodology, validation, analysis, and writing the manuscript. C. Trayford and Dr van Rijt contributed to the validation and analysis. T.M. Shamorkina and Dr Heck have contributed to the methodology, validation, and analysis of the proteomics data. All authors have read, edited, and agreed to the published version of the manuscript.

### Sources of Funding

This research received funding from the European Union’s H2020 Research and Innovation Program under Marie S. Curie cofund RESCUE (Regenerative Medicine & Stem Cells in Utrecht) grant agreement no. 801540 and the Wilhelmina’s Children’s Hospital Research Fund. Proteomics analysis was supported by the Dutch Research Council (Nederlandse Organisatie voor Wetenschappelijk Onderzoek, NWO) through the X-omics Road Map program (project 184.034.019).

### Disclosures

None.

### Supplemental Material

Supplemental Methods

Tables S1–S2

Figures S1–S4

Major Resources Table

References 47–50

ARRIVE Checklist

## References

[1] Lee AC Kozuki N Blencowe H Vos T Bahalim A Darmstadt GL Niermeyer S Ellis M Robertson NJ Cousens S. Intrapartum-related neonatal encephalopathy incidence and impairment at regional and global levels for 2010 with trends from 1990. Pediatr Res. 2013;74:50–72. doi: 10.1038/pr.2013.206 24366463 10.1038/pr.2013.206 PMC3873711

[2] Perez A Ritter S Brotschi B Werner H Caflisch J Martin E Latal B. Long-term neurodevelopmental outcome with hypoxic-ischemic encephalopathy. J Pediatr. 2013;163:454–459. doi: 10.1016/j.jpeds.2013.02.003 23498155 10.1016/j.jpeds.2013.02.003

[3] Kariholu U Montaldo P Markati T Lally P Pryce R Teiserskas J Liow N Oliveira V Soe A Shankaran S. Therapeutic hypothermia for mild neonatal encephalopathy: a systematic review and meta-analysis. Arch Dis Child Fetal Neonatal Ed. 2020;105:F225–F228. doi: 10.1136/archdischild-2018-315711 10.1136/archdischild-2018-315711 30567775

[4] Baak LM Wagenaar N van der Aa NE Groenendaal F Dudink J Tataranno ML Mahamuud U Verhage CH Eijsermans RMJC Smit LS. Feasibility and safety of intranasally administered mesenchymal stromal cells after perinatal arterial ischaemic stroke in the Netherlands (PASSIoN): a first-in-human, open-label intervention study. Lancet Neurol. 2022;21:528–536. doi: 10.1016/S1474-4422(22)00117-X 35568047 10.1016/S1474-4422(22)00117-X

[5] Donega V Van Velthoven CTJ Nijboer CH Van Bel F Kas MJH Kavelaars A Heijnen CJ. Intranasal mesenchymal stem cells treatment for neonatal brain damage: long-term cognitive and sensorimotor improvement. PLoS One. 2013;8:e51253. doi: 10.1371/journal.pone.0051253 23300948 10.1371/journal.pone.0051253 PMC3536775

[6] Donega V Nijboer CH van Tilborg G Dijkhuizen RM Kavelaars A Heijnen CJ. Intranasally administered mesenchymal stem cells promote a regenerative niche for repair of neonatal ischemic brain injury. Exp Neurol. 2014;261:53–64. doi: 10.1016/j.expneurol.2014.06.009 24945601 10.1016/j.expneurol.2014.06.009

[7] Robertson NJ Meehan C Martinello KA Avdic-Belltheus A Boggini T Mutshiya T Lingam I Yang Q Sokolska M Charalambous X. Human umbilical cord mesenchymal stromal cells as an adjunct therapy with therapeutic hypothermia in a piglet model of perinatal asphyxia. Cytotherapy. 2021;23:521–535. doi: 10.1016/j.jcyt.2020.10.005 33262073 10.1016/j.jcyt.2020.10.005 PMC8139415

[8] Serrenho I Rosado M Dinis A Cardoso CM Grãos M Manadas B Baltazar G. Stem cell therapy for neonatal hypoxic-ischemic encephalopathy: a systematic review of preclinical studies. Int J Mol Sci. 2021;22:1–29. doi: 10.3390/ijms22063142 10.3390/ijms22063142 PMC8003344 33808671

[9] Donega V Nijboer CH Braccioli L Slaper-Cortenbach I Kavelaars A Van Bel F Heijnen CJ. Intranasal administration of human MSC for ischemic brain injury in the mouse: in vitro and in vivo neuroregenerative functions. PLoS One. 2014;9:e112339. doi: 10.1371/journal.pone.0112339 25396420 10.1371/journal.pone.0112339 PMC4232359

[10] Wei N Yu SP Gu X Taylor TM Song D Liu XF Wei L. Delayed intranasal delivery of hypoxic-preconditioned bone marrow mesenchymal stem cells enhanced cell homing and therapeutic benefits after ischemic stroke in mice. Cell Transplant. 2013;22:977–991. doi: 10.3727/096368912X657251 23031629 10.3727/096368912X657251

[11] Yang Y Lee EH Yang Z. Hypoxia-conditioned mesenchymal stem cells in tissue regeneration application. Tissue Eng Part B Rev. 2022;28:966–977. doi: 10.1089/ten.TEB.2021.0145 34569290 10.1089/ten.TEB.2021.0145

[12] Chen J Yang Y Shen L Ding W Chen X Wu E Cai K Wang G. Hypoxic preconditioning augments the therapeutic efficacy of bone marrow stromal cells in a rat ischemic stroke model. Cell Mol Neurobiol. 2017;37:1115–1129. doi: 10.1007/s10571-016-0445-1 27858286 10.1007/s10571-016-0445-1 PMC11482114

[13] Hu Y Chen W Wu L Jiang L Qin H Tang N. Hypoxic preconditioning improves the survival and neural effects of transplanted mesenchymal stem cells via CXCL12/CXCR4 signalling in a rat model of cerebral infarction. Cell Biochem Funct. 2019;37:504–515. doi: 10.1002/cbf.3423 31368195 10.1002/cbf.3423

[14] Jiang RH Wu CJ Xu XQ Lu SS Zu QQ Zhao LB Wang J Liu S Shi HB. Hypoxic conditioned medium derived from bone marrow mesenchymal stromal cells protects against ischemic stroke in rats. J Cell Physiol. 2019;234:1354–1368. doi: 10.1002/jcp.26931 30076722 10.1002/jcp.26931

[15] Perez-Riverol Y Bai J Bandla C García-Seisdedos D Hewapathirana S Kamatchinathan S Kundu DJ Prakash A Frericks-Zipper A Eisenacher M. The PRIDE database resources in 2022: a hub for mass spectrometry-based proteomics evidences. Nucleic Acids Res. 2022;50:D543–D552. doi: 10.1093/nar/gkab1038 34723319 10.1093/nar/gkab1038 PMC8728295

[16] Percie du Sert N Hurst V Ahluwalia A Alam S Avey MT Baker M Browne WJ Clark A Cuthill IC Dirnagl U. The ARRIVE Guidelines 2.0: updated guidelines for reporting animal research. PLoS Biol. 2020;18:e3000410–e3000412. doi: 10.1371/journal.pbio.3000410 32663219 10.1371/journal.pbio.3000410 PMC7360023

[17] Vaes JEG van Kammen CM Trayford C van der Toorn A Ruhwedel T Benders MJNL Dijkhuizen RM Möbius W van Rijt SH Nijboer CH. Intranasal mesenchymal stem cell therapy to boost myelination after encephalopathy of prematurity. Glia. 2021;69:655–680. doi: 10.1002/glia.23919 33045105 10.1002/glia.23919 PMC7821154

[18] Nyalali AMK Leonard AU Xu Y Li H Zhou J Zhang X Rugambwa TK Shi X Li F. CD147: an integral and potential molecule to abrogate hallmarks of cancer. Front Oncol. 2023;13:1238051. doi: 10.3389/fonc.2023.1238051 38023152 10.3389/fonc.2023.1238051 PMC10662318

[19] Jinyu L Shuying W Panchan Z Dan C Chao C Xingyu Y Weiwei C. Bone marrow stromal cell antigen 2(BST2) suppresses the migration and invasion of trophoblasts in preeclampsia by downregulating matrix metallopeptidase 2(MMP2). Bioengineered. 2022;13:13174–13187. doi: 10.1080/21655979.2022.2074712 35635087 10.1080/21655979.2022.2074712 PMC9276030

[20] Gonçalves IG Garcia-Aznar JM. Extracellular matrix density regulates the formation of tumour spheroids through cell migration. PLoS Comput Biol. 2021;17:e1008764. doi: 10.1371/journal.pcbi.1008764 33635856 10.1371/journal.pcbi.1008764 PMC7968691

[21] Chen X Yao N Mao Y Xiao D Huang Y Zhang X Wang Y. Activation of the Wnt/β-catenin/CYP1B1 pathway alleviates oxidative stress and protects the blood-brain barrier under cerebral ischemia/reperfusion conditions. Neural Regen Res. 2024;19:1541–1547. doi: 10.4103/1673-5374.386398 38051897 10.4103/1673-5374.386398 PMC10883507

[22] Martínez-Zárate AD MartíMartnez-Záínez-Vieyra AD Alonso-Rangel I Cisneros L Winder B Cerecedo SJ. Dystroglycan depletion inhibits the functions of differentiated HL-60 cells. Biochem Biophys Res Commun. 2014;448:274–280. doi: 10.1016/j.bbrc.2014.04.110 24792180 10.1016/j.bbrc.2014.04.110

[23] Wang C Wang J Liu H Fu Z. Tumor suppressor DLC-1 induces apoptosis and inhibits the growth and invasion of colon cancer cells through the Wnt/β-catenin signaling pathway. Oncol Rep. 2014;31:2270–2278. doi: 10.3892/or.2014.3057 24604602 10.3892/or.2014.3057

[24] Qing Y Ono T Kohara Y Watanabe A Ogiso N Ito M Nakashima T Takeshita S. Emilin2 marks the target region for mesenchymal cell accumulation in bone regeneration. Inflamm Regen. 2024;44:27. doi: 10.1186/s41232-024-00341-6 38831448 10.1186/s41232-024-00341-6 PMC11145771

[25] Zhou X Li Y Yang C Chen D Wang T Liu T Yan W Su Z Peng B Ren X. Cordycepin reprogramming lipid metabolism to block metastasis and EMT via ERO1A/mTOR/SREBP1 axis in cholangiocarcinoma. Life Sci. 2023;327:121698. doi: 10.1016/j.lfs.2023.121698 37080351 10.1016/j.lfs.2023.121698

[26] Sun Z Qin X Fang J Tang Y Fan Y. Multi-omics analysis of the expression and prognosis for FKBP gene family in renal cancer. Front Oncol. 2021;11:697534. doi: 10.3389/fonc.2021.697534 34476212 10.3389/fonc.2021.697534 PMC8406630

[27] Longstreth JH Wang K. The role of fibronectin in mediating cell migration. Am J Physiol Cell Physiol. 2024;326:C1212–C1225. doi: 10.1152/ajpcell.00633.2023 38372136 10.1152/ajpcell.00633.2023

[28] Chen H Wang SH Chen C Yu XY Zhu JN Mansell T Novakovic B Saffery R Baker PN Han TL. A novel role of FoxO3a in the migration and invasion of trophoblast cells: from metabolic remodeling to transcriptional reprogramming. Mol Med. 2022;28:92. doi: 10.1186/s10020-022-00522-4 35941589 10.1186/s10020-022-00522-4 PMC9358829

[29] Schneider L Liu J Zhang C Azoitei A Meessen S Zheng X Cremer C Gorzelanny C Kempe-Gonzales S Brunner C. The role of interleukin-1-receptor-antagonist in bladder cancer cell migration and invasion. Int J Mol Sci. 2021;22:5875. doi: 10.3390/ijms22115875 34070905 10.3390/ijms22115875 PMC8198563

[30] Yoon S Leube RE. Keratin intermediate filaments: intermediaries of epithelial cell migration. Essays Biochem. 2019;63:521–533. doi: 10.1042/EBC20190017 31652439 10.1042/EBC20190017

[31] Xiao Q Ge G. Lysyl oxidase, extracellular matrix remodeling and cancer metastasis. Cancer Microenviron. 2012;5:261–273. doi: 10.1007/s12307-012-0105-z 22528876 10.1007/s12307-012-0105-z PMC3460045

[32] Lourenco S Teixeira VH Kalber T Jose RJ Floto RA Janes SM. Macrophage migration inhibitory factor–CXCR4 is the dominant chemotactic axis in human mesenchymal stem cell recruitment to tumors. J Immunol. 2015;194:3463–3474. doi: 10.4049/jimmunol.1402097 25712213 10.4049/jimmunol.1402097 PMC4374168

[33] Toss MS Miligy IM Gorringe KL AlKawaz A Khout H Ellis IO Green AR Rakha EA. Prolyl-4-hydroxylase Α subunit 2 (P4HA2) expression is a predictor of poor outcome in breast ductal carcinoma in situ (DCIS). Br J Cancer. 2018;119:1518–1526. doi: 10.1038/s41416-018-0337-x 30410060 10.1038/s41416-018-0337-x PMC6288166

[34] Kopsida M Liu N Kotti A Wang J Jensen L Jothimani G Hildesjo C Haapaniemi S Zhong W Pathak S. RhoB expression associated with chemotherapy response and prognosis in colorectal cancer. Cancer Cell Int. 2024;24:75. doi: 10.1186/s12935-024-03236-1 38355625 10.1186/s12935-024-03236-1 PMC10867990

[35] Liu H Wang N Li J Wang W Han W Li Q. AAV1-mediated shRNA knockdown of *SASH1* in rat bronchus attenuates hypoxia-induced pulmonary artery remodeling. Hum Gene Ther. 2021;32:796–805. doi: 10.1089/hum.2020.242 33297837 10.1089/hum.2020.242

[36] Tang N Mack F Haase VH Simon MC Johnson RS. pVHL function is essential for endothelial extracellular matrix deposition. Mol Cell Biol. 2006;26:2519–2530. doi: 10.1128/MCB.26.7.2519-2530.2006 16537898 10.1128/MCB.26.7.2519-2530.2006 PMC1430327

[37] Gurney ME Heinrich SP Lee MR Yin HS. Molecular cloning and expression of neuroleukin, a neurotropbic factor for spinal and sensory neurons. Science. 1986;234:566–574. doi: 10.1126/science.3764429 3764429 10.1126/science.3764429

[38] Gonthier B Nasarre C Roth L Perraut M Thomasset N Roussel G Aunis D Bagnard D. Functional interaction between matrix metalloproteinase-3 and semaphorin-3C during cortical axonal growth and guidance. Cereb Cortex. 2007;17:1712–1721. doi: 10.1093/cercor/bhl082 17021275 10.1093/cercor/bhl082

[39] Niu S Renfro A Quattrocchi CC Sheldon M D’arcangelo G. Reelin promotes hippocampal dendrite development through the VLDLR/ApoER2-Dab1 pathway. Neuron. 2004;41:71–84. doi: 10.1016/s0896-6273(03)00819-5 14715136 10.1016/s0896-6273(03)00819-5

[40] Ock J Wu J Liu FY Fridayana FR Niloofar L Nhat VM Hong SS Kang JH Suh JK Yin GN. Heme-binding protein 1 delivered via pericyte-derived extracellular vesicles improves neurovascular regeneration in a mouse model of cavernous nerve injury. Int J Biol Sci. 2023;19:2663–2677. doi: 10.7150/ijbs.81809 37324943 10.7150/ijbs.81809 PMC10266087

[41] Wang SN Miao CY. Targeting NAMPT as a therapeutic strategy against stroke. Stroke Vasc Neurol. 2019;4:83–89. doi: 10.1136/svn-2018-000199 31338216 10.1136/svn-2018-000199 PMC6613878

[42] Prasad CP Gogia A Batra A. Essential role of aerobic glycolysis in epithelial-to-mesenchymal transition during carcinogenesis. Clin Transl Oncol. 2022;24:1844–1855. doi: 10.1007/s12094-022-02851-6 35751743 10.1007/s12094-022-02851-6

[43] Braga CL da Silva LR Santos RT de Carvalho LRP Mandacaru SC de Oliveira Trugilho MR Rocco PRM Cruz FF Silva PL. Proteomics profile of mesenchymal stromal cells and extracellular vesicles in normoxic and hypoxic conditions. Cytotherapy. 2022;24:1211–1224. doi: 10.1016/j.jcyt.2022.08.009 36192337 10.1016/j.jcyt.2022.08.009

[44] Riis S Stensballe A Emmersen J Pennisi CP Birkelund S Zachar V Fink T. Mass spectrometry analysis of adipose-derived stem cells reveals a significant effect of hypoxia on pathways regulating extracellular matrix. Stem Cell Res Ther. 2016;7:52. doi: 10.1186/s13287-016-0310-7 27075204 10.1186/s13287-016-0310-7 PMC4831147

[45] Liu J He J Huang Y Ge L Xiao H Zeng L Jiang Z Lu M Hu Z. Hypoxia-preconditioned mesenchymal stem cells attenuate microglial pyroptosis after intracerebral hemorrhage. Ann Transl Med. 2021;9:1362. doi: 10.21037/atm-21-2590 34733914 10.21037/atm-21-2590 PMC8506532

[46] Tajiri N Duncan K Antoine A Pabon M Acosta SA de la Pena I Hernadez-Ontiveros DG Shinozuka K Ishikawa H Kaneko Y. Stem cell-paved biobridge facilitates neural repair in traumatic brain injury. Front Syst Neurosci. 2014;8:116. doi: 10.3389/fnsys.2014.00116 25009475 10.3389/fnsys.2014.00116 PMC4068001

[47] Schweppe DK Eng JK Yu Q Bailey D Rad R Navarrete-Perea J Huttlin EL Erickson BK Paulo JA Gygi SP. Full-featured, real-time database searching platform enables fast and accurate multiplexed quantitative proteomics. J Proteome Res. 2020;19:2026–2034. doi: 10.1021/acs.jproteome.9b00860 32126768 10.1021/acs.jproteome.9b00860 PMC7295121

[48] Tyanova S Temu T Sinitcyn P Carlson A Hein MY Geiger T Mann M Cox J. The Perseus computational platform for comprehensive analysis of (prote)omics data. Nat Methods. 2016;13:731–740. doi: 10.1038/nmeth.3901 27348712 10.1038/nmeth.3901

[49] Sherman BT Hao M Qiu J Jiao X Baseler MW Lane HC Imamichi T David CW. DAVID: a web server for functional enrichment analysis and functional annotation of gene lists (2021 update). Nucleic Acids Res. 2022;50:W216–W221. doi: 10.1093/nar/gkac194 35325185 10.1093/nar/gkac194 PMC9252805

[50] Qureshi OS Bon H Twomey B Holdsworth G Ford K Bergin M Huang L Muzylak M Healy LJ Hurdowar V. An immunofluorescence assay for extracellular matrix components highlights the role of epithelial cells in producing a stable, fibrillar extracellular matrix. Biol Open. 2017;6:1423–1433. doi: 10.1242/bio.025866 29032370 10.1242/bio.025866 PMC5665462

